# *Cryptococcus neoformans* Meningoencephalitis in an Immunocompetent Patient after COVID-19 Infection

**DOI:** 10.1155/2021/5597473

**Published:** 2021-06-04

**Authors:** Hebah Ghanem, Geetha Sivasubramanian

**Affiliations:** Division of Infectious Diseases, Department of Internal Medicine, University of California, San Francisco, Fresno, California, USA

## Abstract

*Cryptococcus neoformans* is a saprophytic fungus that causes fatal disseminated infections in immunocompromised hosts. Since the onset of the COVID-19 pandemic, multiple cases of secondary viral, bacterial, and fungal infections have been reported in patients after SARS-CoV-2 infection. We describe here a case of severe cryptococcal meningitis that developed in a previously healthy patient one week after treatment of SARS-CoV-2 infection with dexamethasone. This case adds to the growing knowledge of emerging secondary infectious complications including opportunistic pathogens after SARS-CoV-2 infection. While few reports allude to depressed T-cell function and lymphopenia due to SARS-CoV-2 infection, further studies are needed to evaluate the effects of this infection and its treatment on the immune system and its contribution to the emergence of secondary opportunistic infections.

## 1. Introduction

*Cryptococcus neoformans* is an opportunistic fungal pathogen [1]. In immunocompromised patients such as those with HIV/AIDS, transplant recipients, and patients with liver cirrhosis, it can cause devastating meningoencephalitis [2]. In the current COVID-19 pandemic, there have been many observational and retrospective studies reporting the association of COVID-19 infection and other bacterial, viral, and rarely fungal infections, such as streptococcus pneumonia, mycoplasma, rhinovirus, influenza, and aspergillosis [3]. There have been 2 reports so far of cryptococcemia, with blood cultures growing the fungus in COVID-19 patients, one in a transplant recipient, and other in a patient who received tocilizumab treatment for COVID-19-induced cytokine storm [4, 5]. In both these reports, blood cultures were positive for *Cryptococcus*, but meningoencephalitis was not documented. An underlying immunosuppressive medical condition was present in one of them. We present here a previously unreported case of cryptococcal meningoencephalitis in a patient with SARS-CoV-2 infection treated with dexamethasone in an otherwise healthy elderly patient.

## 2. Case Presentation

A 73-year-old Caucasian woman with no known previous medical conditions was transferred to our facility for management of hydrocephalus. Approximately 2 weeks prior to this, she was admitted to an outside hospital after she sustained a fall and developed right hip fracture. She underwent right hip hemiarthroplasty. Postoperatively, she developed fever and mild hypoxia requiring supplemental oxygen. A chest X-ray showed patchy bilateral infiltrates, and she tested positive for SARS-CoV-2 infection. She was treated with a 5-day course of azithromycin and Dexamethasone 6 mg daily to complete a 10-day course. A week after this, she was noticed to have new neurological symptoms and signs with unsteady gait, frequent falls, and complete aphasia. A contrast-enhanced computed tomography scan of the head at this point showed new-onset hydrocephalus for which she was transferred to our facility for higher level of care. Due to progressive obtundation, she was intubated for airway protection. Her labs upon arrival showed normal total white blood cell count, although the absolute lymphocyte count was diminished at 700 per microliter liver and renal functions. HIV serology was nonreactive. Her liver and kidney functions were normal.

She underwent an emergent placement of external ventricular drain (EVD) to relieve intracranial pressure for impending herniation. Cerebrospinal fluid (CSF) analysis revealed white blood cell count of 25 cells/cubic mm, protein 173 gm/dl, and glucose less than 10 mg/dl. CSF cryptococcal antigen was positive, and later cultures also grew *Cryptococcus neoformans*. In retrospect, the patient's family was questioned about any known potential exposures, but none was apparent. She was started on induction therapy with intravenous amphotericin B along with flucytosine. She needed multiple lumbar punctures, lumbar drain, and eventually had a ventriculoperitoneal shunt placed. CSF cultures were sterilized after 13 days of induction therapy. She remained ventilator dependent and developed ventilator-associated pneumonia due to extended-spectrum *Escherichia coli*. This was treated with a 7-day course of meropenem. She had tracheostomy and gastrostomy tube placement and eventually discharged to a rehabilitation facility.

## 3. Discussion

Cryptococcosis is a fungal infection caused by *Cryptococcus* species, *Cryptococcus neoformans* and *Cryptococcus gattii.* They are ubiquitous in the environment, especially in soil enriched with avian feces, particularly chicken and pigeon droppings [1]. Inhalation is the usual route of infection. Immunocompromised patients, especially patients with impaired cell-mediated immunity such as transplant recipients and those with HIV/AIDS, are prone to more serious infection and rapid progression with dissemination [2]. Meningoencephalitis is the most common presentation of disseminated infection. Knowledge about overall coinfections in patients with COVID-19 infection is limited to case reports at present. Infections due to bacteria such as *Staphylococcus aureus*, *Streptococcus pneumonia*e, *Klebsiella pneumoniae*, *Mycoplasma pneumonia*, chlamydia, fungi such as candida and aspergillus, and viruses such as influenza rhinovirus and parainfluenza have been reported [3].

Coinfection of SARS-CoV-2 and cryptococcal infection has been only rarely reported. We did literature review using search engines PubMed and Cochrane using key words COVID-19, infection, SARS-Cov-2, *Cryptococcus neoformans*, and cryptococcus meningitis and identified two such reports. Passarelli et al. reported case of positive SARS-CoV-2 in an ascitic fluid from a kidney transplant patient with decompensated cirrhosis and COVID-19 who also developed fungemia due to *Cryptococcus neoformans* [4]. Khatib et al. reported a case of cryptococcemia in a patient who received tocilizumab for COVID-19, and in their paper, they were urging clinicians to balance the use of immunosuppressant drugs and tocilizumab for COVID-19 patients to avoid the development of infections such as cryptococcemia [5]. Studies have revealed several predisposing conditions for cryptococcal meningitis including HIV infection, transplant recipient status, malignancies, and steroid use [6]. A depressed T-cell response seems to be the underlying driving factor in these conditions. Recent studies reported that SARS-CoV-2 infection may affect T-cell response in multiple ways such as by causing lymphopenia, with reduction in CD4+ and CD8+ T cells as well as reducing Interferon-*γ* production by CD4+ T cells [7–9]. Qin et al. studied T-cell responses in 452 patients with COVID-19 in Wuhan, China. Lymphocyte subsets were analyzed in 44 patients with COVID-19. The total number of B cells, T cells, and NK cells significantly decreased in patients with COVID-19 and more evident in the severe cases compared to the nonsevere group. They found that the number of T cells significantly decreased and more hampered in severe cases. Both helper T cells and suppressor T cells in patients with COVID-19 were below normal levels. Similar findings were noted in a study by Chen et al., who also found that reduction in interferon-*γ* production by CD4 T cells was impaired in patients with severe SARS-CoV-2 infection.

Although corticosteroid use may have contributed to T-cell dysfunction in our patient, the role of T-cell immune dysfunction directly due to SARS-CoV-2 infection in the pathogenesis is a possibility. Our case highlights that invasive cryptococcal meningitis could present as a secondary infection complicating SARS-Cov-2 infection. The development of severe disseminated cryptococcal infection in this setting suggests further studies are needed to study the impact of SARS-CoV-2 infection on T-cell function, especially with added risks from therapeutic modalities such as corticosteroids.

## 4. Conclusions

The knowledge about the extent to which COVID-19 dysregulates the immune system is still evolving. Some published reports point towards impaired T-cell function in these patients. With the added risk due to immunosuppressive therapies such as corticosteroids, these patients are at risk of secondary fungal infections. We report a case of severe cryptococcal meningitis in a previously apparently healthy patient after being diagnosed with COVID-19 infection and treated with corticosteroids. Further studies are needed to characterize the effect of SARS-CoV-2 infection on host immune response, and continued vigilance for development of secondary opportunistic infections in such patients is essential.
